# Impact of linker histone in the formation of ambochlorin-calf thymus DNA complex: Multi-spectroscopic, stopped-flow, and molecular modeling approaches

**DOI:** 10.22038/IJBMS.2021.58829.13070

**Published:** 2021-11

**Authors:** Azam Askari, Parisa Mokaberi, Maryam Dareini, Morvarid Medalian, Mahtab Pejhan, Maryam Erfani, Maryam Asadzadeh-Lotfabad, Mohammad Reza Saberi, Jamshidkhan Chamani

**Affiliations:** 1 Department of Biology, Faculty of Sciences, Mashhad Branch, Islamic Azad University, Mashhad, Iran; 2 Medical Chemistry Department, School of Pharmacy, Mashhad University of Medical Sciences, Mashhad, Iran

**Keywords:** Calf thymus DNA, Linker histone, Molecular modeling, Spectroscopy, Circular dichroism

## Abstract

**Objective(s):**

This study aimed to evaluate the role of the linker histone (H_1_) in the binding interaction between ambochlorin (Amb), and calf thymus DNA (ctDNA) as binary and ternary systems.

**Materials and Methods:**

The project was accomplished through the means of absorbance, fluorescence, stopped-flow circular dichroism spectroscopy, viscosity, thermal melting, and molecular modeling techniques.

**Results:**

Spectroscopic analysis revealed that although Amb was strongly bound to both ctDNA and ctDNA-H_1_, it showed a greater tendency to ctDNA in the presence of the linker histone. The obtained thermodynamic parameters revealed that both Amb-ctDNA and Amb-ctDNA-H_1_ interactions were spontaneous, endothermic, and entropy-favored, and hydrophobic interactions played the main role in the formation and stabilization of complexes. Analysis of the stopped-flow circular dichroism results revealed that the binding process of Amb-ctDNA and Amb-ctDNA-H_1_ required a time of more than 150 milliseconds to complete. Moreover, Amb-ctDNA complex formation was marginally decelerated in the presence of the linker histone. The docking results suggested that the presence of the linker histone may alter the binding sites of Amb from ctDNA minor grooves to major grooves.

**Conclusion:**

All quenching processes were governed by a dynamic mechanism. Additionally, Amb did not stabilize or induce considerable conformational changes in ctDNA and ctDNA-H_1_ complex upon binding. In silico molecular docking results confirmed that Amb was bound to the double-helical ctDNA and ctDNA-H_1_ via ctDNA grooves. In summary, some binding properties of the interactions between Amb and ctDNA change in the presence of the linker histone.

## Introduction

DNA is a long partially flexible biopolymer that contains the detailed biological information required for cell subsistence. This adaptable molecule is one of the most biologically researched molecules. DNA is the focus of attention for designing various classes of medications, such as anticancer, antibiotic, and antiviral drugs. This pharmacological interest is based on the fact that drug molecules can target DNA and influence it functionally or structurally. The induced changes in the structure or functions of DNA molecules can extend their influence on various aspects of cell life. Up to now, numerous researches have been directed towards deciphering the characteristics of binding interactions between various drugs and DNA. This subject is now one of the central and fundamental topics in the fields of medicine, chemistry, and biology (1, 2). In the light of the provided information regarding the DNA-drug interactions, we can advance our understanding of the mechanism of action, therapeutic efficiency, and possible side effects of such compounds. Moreover, these findings provide practical information efficiently used for the rational designing of novel drugs. 

In almost all eukaryotic nuclei, DNA molecules are highly condensed and organized as a nucleoprotein complex named chromatin, which is composed of a DNA molecule covered and interacting with some types of nuclear proteins (3, 4). The chromatin proteins are known to play structural or functional roles. The most important structural proteins are a family of highly charged proteins with rather low molecular weight named histones. This family of chromatin proteins binds to DNA and bends it to be perfectly fit into the nucleolus (5). Histones are classified into core or octamer (i.e., H_2_, H_3_, and H_4_) and the linker histone (H_1_). The linker histone is longer than octamer histones and is regarded as a crucial and relatively dynamic part of chromatin. This class of histone proteins significantly contributes to the higher level of chromatin compaction. In chromatin structure, one DNA molecule winds around a protein complex comprising eight molecules of core histones. The resultant structure, named nucleosome, is the repeating unit and basic structure of chromatin (6, 7). In order to stabilize the nucleosomal structure, one copy of the linker histone binds to both nucleosomal core and spacer DNA. It is worth mentioning that although the linker histone is generally equivalent to H_1_, in certain types of cells it is exchanged by a rare form of histone proteins, named H_5_, which functions the same as H_1_.

Small-molecule cancer drugs and monoclonal antibodies are the two principal classes of chemotherapy drugs vastly used in cancer targeted therapy (8). Considering that small-molecule drugs have a molecular weight below 500 Da (9), they can easily pass through the cell membrane and nuclear envelope. Hence, these agents can bind to various macromolecules inside the cell and nucleus, such as DNA, RNA, and protein molecules. These binding interactions can be made by either covalent bonds or non-covalent forces. It is also possible that both of the covalent and non-covalent interactions contribute to a complex formed between bio-macromolecules and small-molecule drugs. Whereas the covalent binding interactions are strong and irreversible, non-covalent interactions are reversible and comparatively weaker. The reversibility of the non-covalent DNA binders made them preferable in designing drugs. The binding mechanism of the non-covalent agents to DNA is classified into three main modes: intercalation, groove binding, and outside or external binding. The non-covalent binding agents can interact with DNA either in one of the above-mentioned models or more than one model. It is assumed that this feature is mostly related to the therapeutic efficiency and mechanism of action of such compounds (10). It should be noted that some DNA intercalating or groove-binding agents can bind to DNA covalently as well. Some of the DNA-targeting drugs can also bind to other biological molecules or complexes, such as RNA, proteins, and enzymes. One example of such compounds is cisplatin, an anti-neoplastic drug, which can bind to both DNA molecules and ribosomes (11). In addition to different mechanisms of binding, small-molecule drugs may exhibit distinctive sequential preferences to DNA. This feature which is mostly resulted from the slight differences in the binding mechanisms gives rise to diverse pharmacological and cytotoxic effects (12).

DNA-binding intercalators reversibly unwind DNA to be set between base pairs. These interactions normally stabilize and lengthen DNA. The extent of DNA unwinding induced by the intercalation binding mode is widely variable and depends on the characteristics of the binding agent. The DNA groove-binding agents can interact with either minor or major grooves of DNA. Although small-molecule drugs mainly prefer minor grooves, some molecules such as most DNA binding proteins bind to the major grooves. Outside-binding agents bind to the phosphates of the DNA backbone electrostatically through positively charged groups of their molecules. In the intercalation mode, the binding agents are relatively protected from solution, and thus, not easily affected by the surrounding changes. In contrast, when a molecule binds to DNA via grooves or phosphates of DNA, it will be exposed to environmental changes by a considerable amount. This is due to the fact that the binding agents are much more accessible to the solution compared with intercalators.

From one standpoint, small-molecule drugs that target DNA are classified under the three following classes: 1) molecules that merely bind to naked DNA, 2) those that can only bind to chromosomal DNA, and 3) those that have the ability to bind to the naked DNA as well as chromosomal DNA with similar or different affinities. To put it another way, the presence of structural chromatin proteins, especially histones, can notably affect the way small molecules bind to DNA. It is thus clear that evaluation of the DNA binding interactions in the presence of the histone proteins provides detailed insights into DNA binding interactions.

**Figure 1:**
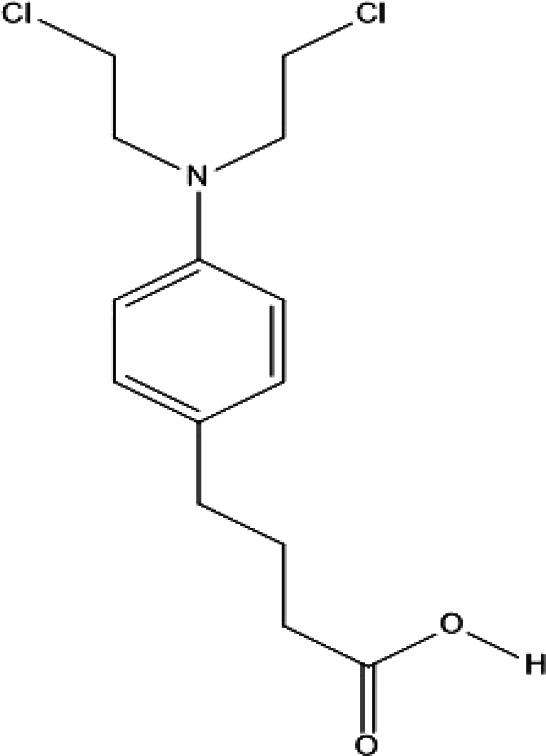
Molecular structure of the ambochlorin(4-[4-[bis(2-chloroethyl)amino]phenyl]butanoic acid)

Ambochlorin (Figure 1), known as chlorambucil and Leukeran, is a clinically approved small-molecule cancer drug mostly taken during the treatment process of chronic lymphocytic leukemia (13). Previous *in vitro* experiments have indicated that Amb can bind to the naked DNA and RNA molecules (14-16). Furthermore, earlier studies revealed that the drug can be a potential substrate for the Pi class of Glutathione-S-transferase enzymes (17). However, no detailed characteristics of the binding interaction between Amb and DNA in the presence of histone have hitherto been reported. 

The purpose of this study is to investigate the binding interaction between Amb and ctDNA. Furthermore, the role of the linker histone in this binding interaction was evaluated. The assumption that the presence of the linker histone may affect the binding interaction between ctDNA and Amb was mainly based on the fact that the binding interaction between H_1_ and DNA makes DNA less accessible and thus predominantly inactive (18). In order to meet the goal of this study, several spectroscopic techniques including UV-visible, fluorescence, and circular dichroism spectroscopies were employed. Further investigations were obtained using thermal denaturation and viscometric studies. A more profound understanding of the binding interactions was provided by analyzing the kinetics of interactions using the stopped-flow circular dichroism technique. Additionally, with the aim of predicting and achieving an in-depth understanding of Amb-ctDNA interaction in the presence and absence of the linker histone, *in silico* molecular modeling was applied as well.

## Materials and Methods

***Reagents***

Ambochlorin, calf thymus DNA, linker histone protein, ethidium bromide, and acridine orange were all purchased from Sigma Aldrich, USA. The chemicals were kept at 4 °C and used without further purification. The UV absorption of the purchased ctDNA at 260 and 280 nm was measured. Since the ratio of A_260 _to A_280 _was higher than 1.8, we deduced that the purity of ctDNA was sufficiently adequate. A buffer of Tris-HCl 10 mM at pH 6.8 was utilized for preparing all solutions excluding sodium chloride (NaCl) and potassium iodide (KI) solutions, which were prepared by dissolving the salts in ultra-pure water. The pH of the Tris-HCl buffer was adjusted using NaOH.

***Preparation of solutions***

The concentration of ctDNA solution was determined by using Beer-Lambert`s equation on the basis of ultraviolet absorption of the solution containing ctDNA 0.01%. The ligand solution (0.07 mM) was synthesized by dissolving an appropriate amount of Amb in Tris-HCl buffer 10 mM. To prepare ctDNA-H_1 _solution a ratio of 1:1 of ctDNA and linker histone solutions with the same concentration (1.3×10^-4^M) was used. The concentrations of KI and NaCl used for the ionic strength studies were 76.1 mM and 1 M, respectively. For the fluorescence replacement studies, EB (6.7×10^-3^ M) and AO (4.2×10^-8^ M) were used as the fluorescent intercalator probes.

***Apparatus and methods***

In order to reach greater validity and reliability in the experimental data, we repeated all the experiments 3 times and reported the averaged values.

*Fluorescence quenching and RLS studies*

To perform the emission and RLS studies a Hitachi F-2500 spectrophotometer was used. The fluorescence spectroscopic examinations were run in a transparent quartz sample cell of 1 cm path length. In the fluorescence quenching studies, the samples were excited at the wavelength of 323 nm and the spectra were recorded over wavelengths from 320 to 800 nm. For recording the RLS spectra, both the excitation and emission monochromators of the F-2500 Hitachi spectrophotometer were scanned in the synchronous scan mode (Δλ=0 nm). The excitation wavelength was set at 220 nm and the spectra were recorded over the wavelengths from 220 to 600 nm. In both fluorescence quenching and RLS studies, the spectra of Amb (0.07 mM) were recorded in the absence and presence of ctDNA and ctDNA-H_1_ complex (in the concentration range of 0 to 1× 10^-6^ mM). 

*Competition experiments*

The fluorescence competition displacement studies were recorded with the F-2500 spectrophotometer by using the fluorescence probes AO and EB. Amb solution (0.07 mM) was gradually added to a fluorescence quartz cuvette containing EB-ctDNA and AO-ctDNA complexes and the fluorescence intensities of the samples were measured when the concentration of Amb in the observation cell increased from 0 to 0.04 mM. The excitation wavelengths for the solutions containing EB and AO were fixed at 440 and 490 nm, respectively. In the case of ctDNA-H_1_, a solution containing ctDNA-H_1_ was used as an alternative to ctDNA. It should be noted that in all quenching, competition, and RLS spectroscopic experiments, a time interval of 3 min between the injection and spectra recording was applied.

*Ionic strength studies*

The effect of ionic intensity on the binding interactions was evaluated using the ionic solutions of NaCl (1 M) and KI (76.1 mM). The small aliquots of NaCl were titrated into an absorption quartz cuvette containing a fixed concentration of Amb-ctDNA and Amb-(ctDNA-H_1_) complexes and the changes of the absorption spectra through the titration were monitored. To evaluate the accuracy of the obtained results, another series of tests was then performed using KI as the replacement of NaCl. 

*Thermal denaturation studies*

To carry out DNA denaturation investigations, the UV absorbance of ctDNA (1.3×10^-4^ M) at its maximum absorption wavelength both in the absence and presence of Amb (0.07 mM) was measured. Each sample was gently warmed up from 20 to 80 °C. A thermocouple-equipped V-630 Jasco spectrophotometer was employed and the data were gathered every 2 °C. The results were visualized by plotting the relative absorbance intensity of ctDNA (or ctDNA-Amb) versus temperature. The melting temperatures (T_m_) were estimated from points of inflection of the derivative denaturation profile (19). In the study of the linker histone, a solution containing the ctDNA-H_1_ complex was used.

*Viscosity measurements*

Viscometry studies were conducted using a Haake Ostwald viscometer. The viscosities of the constant concentrations of ctDNA and ctDNA-H_1_ solutions (1.3×10^-4^ M) were measured by adding various concentrations of Amb. In order to accurately measure the flow time of the samples, a sensitive stopwatch was utilized. The relative viscosity was recorded after each drug injection and the results were presented by plotting (η/η_0_)^1/3 ^versus (3) / [ctDNA] or (3)/[ctDNA-H_1_] in which η and η_0_ are the viscosities of ctDNA or ctDNA-H_1_ in the presence and absence of the Amb, respectively. 

*Circular dichroism (CD) studies*

To elucidate the conformational effects of Amb on ctDNA and ctDNA-H_1_ complex, CD spectroscopy studies were performed using a J-815 Jasco spectropolarimeter. The UV-CD spectra of ctDNA and ctDNA-H_1_ were recorded in the absence and presence of increasing concentration of Amb on a quartz cuvette with a 1-cm path length. All the CD spectra were measured over the wavelengths from 230 to 300 nm. The CD spectrum of Tris-HCl buffer 10 mM was recorded and subtracted from the samples. The results of CD spectroscopy experiments were visualized by plotting the ellipticity (mdeg) versus wavelength (nm).

*Stopped-flow circular dichroism (stopped-flow CD) studies*

Stopped-flow CD experiments were performed using the J-815 spectropolarimeter equipped with a stopped-flow apparatus. Since kinetic constants are temperature-dependent, the temperature of samples was kept constant at room temperature using a circulating water bath. The solutions of ctDNA or ctDNA-H_1_ (1.3×10^-4 ^M) and Amb (0.07 mM) were flowed into the mixing chamber with a mixing ratio of 1:1. When the flows were stopped, the interactions were rigorously monitored by the CD spectropolarimeter. Each experiment was repeated 5 times and the averaged value was reported. The obtained data were presented by plotting the changes of ellipticity (mdeg) versus time.

*Comparing the interaction of Amb with single-strand and double-strand ctDNA*

The single-strand ctDNA (ss ctDNA) was derived from double-strand ctDNA (ds ctDNA) by heating native ctDNA molecules in a boiling-water bath. Then, the sample cooled promptly by using a water-ice bath. The fluorescence intensity of Amb (0.07 mM) was recorded upon addition of ss ctDNA or ds ctDNA (1.3 × 10^-4^). The obtained data were represented graphically by plotting relative fluorescence intensities of Amb versus [ss ctDNA] or [ds ctDNA]. The ssctDNA-H_1_ complex was obtained by adding the linker histone solutions to a cuvette containing ss ctDNA solution by a ratio of 1:1. Similar experiments were then conducted by adding ss ctDNA-H_1_ and ds ctDNA-H_1_ to a fluorescence cuvette containing Amb.

*Molecular modeling studies*

The Molecular Operating Environment (MOE) software (version 2008.10) was used to simulate the interactions. The crystal structures of ctDNA and linker histone were obtained from the protein data bank with PDB IDs of 1BNA and 5j8j, respectively. The base pair sequence of the obtained DNA molecule was d (CpGpCpGpApApTpTpCpGpCpG). The chemical structure of the ligand was downloaded from DrugBank (id: DB00291). To obtain the structure of the ctDNA-H_1_ complex, PDB-format files of ctDNA and linker histone were docked using the Hex 8.0.0 program and then optimized by energy minimization. The chemical structure of the ligand and receptors were docked by the MOE program. The receptor and ligand molecules were then protonated by adding hydrogen atoms on the basis of their standard geometry at 300 K and pH 7. The docking procedures were then carried out using triangle matcher placement and scored by London dG. To attain the most optimized conformer, docking protocol was permitted to rotate all rotatable bonds of ligand. Furthermore, to enhance the quality of docking results, refinement was employed by using force field placement with London dg scoring.

## Results

***Fluorescence quenching measurements***

**Figure 2:**
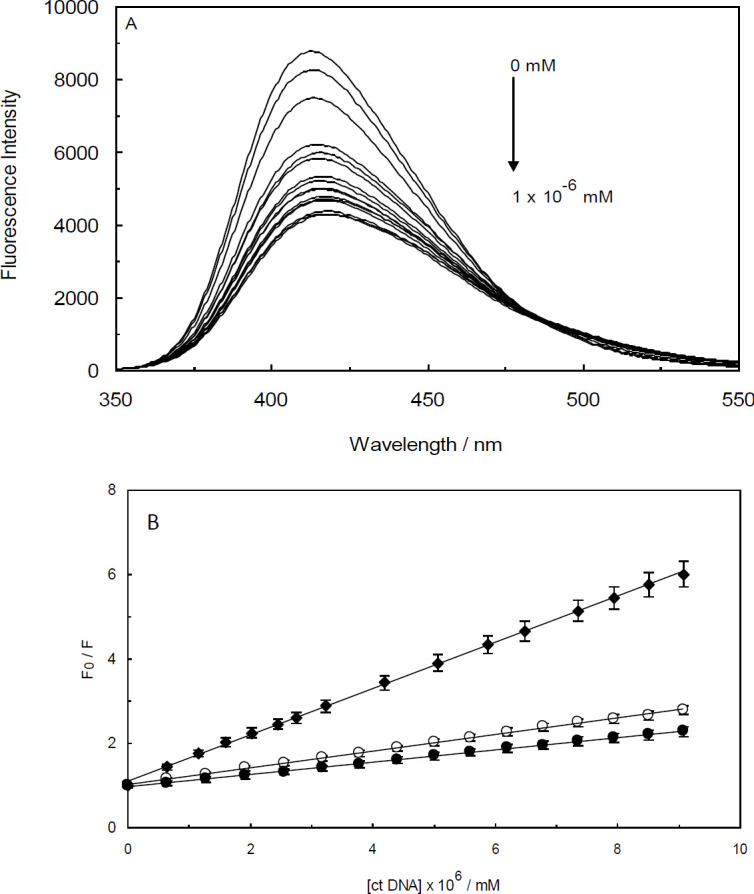
(A) Fluorescence spectra of Ambochlorin (Amb) (0.07 mM) in the presence of different concentrations of ctDNA (0 - 1 × 10-6 mM) at T=298 K and pH 6.8. (B) Stern-Volmer plots for Amb-ctDNA complex at 298 (closed circles), 303 (open circles) and 308 K (closed diamond)

**Figure 3:**
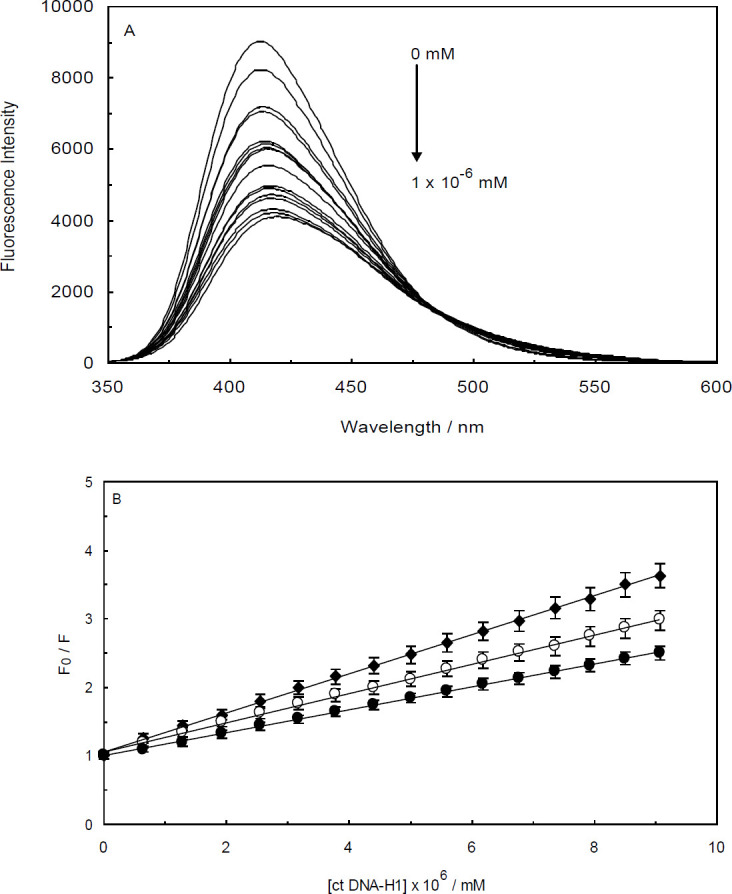
(A) Fluorescence spectra of Ambochlorin (Amb) (0.07 mM) in the presence of different concentration of ctDNA-H1 complex in the concentration range of 0 to 1×10-6mM; T=298K; pH 6.8. (B) Stern-Volmer plots for the Amb-(ctDNA-H1) complex at 298(closed circles), 303 (open circles) and 308K (closed diamond)

With the aim of investigating the interaction between the drug and ctDNA, the fluorescence intensity of Amb was measured in the presence of various concentrations of ctDNA and ctDNA-H_1_ complex. Amb represented a peak of maximum emission around 412 nm. The intrinsic fluorescence emission of Amb should be attributable to its aromatic ring (Figure 1). It is apparent from Figure 2A that the gradual addition of ctDNA results in significant quenching of Amb emission intensity. The observed quenching effect should be due to the formation of a complex between Amb and ctDNA. Similar behavior was observed by the addition of the ctDNA-H_1_ complex to Amb solution (Figure 3A). The quenching processes were further analyzed by using the Stern-Volmer equation:

F_0_/F = 1+ K_SV _[Q]                                      (1)

where, F_0 _and F are the fluorescence intensities of Amb in the absence and presence of quenchers (ctDNA or ctDNA-H_1_), respectively. [Q] is the concentration of ctDNA or ctDNA-H_1_ as quenchers and K_sv_ is referred to as the Stern–Volmer quenching constant. According to equation (1), the quenching process and Stern-Volmer constant are not dependent on fluorophore concentration and only vary by the quencher concentration. Plotting the Stern-Volmer`s equation allows us to calculate the values of Ksv from the slope. A more sensitive interaction represents a steeper slope in the related Stern-Volmer plot and consequently a larger value for K_SV_.

Measuring the value of K_SV _is helpful in many areas, such as impurity quenching, photochemical and bimolecular reaction rates, and energy transfer (20). It also supplies valuable details on the nature of DNA-ligand interaction. Under our experimental conditions, the values of K_SV_ for Amb-ctDNA and Amb-ctDNA-H_1_ interactions were 1.46×10^8^ and 1.67×10^8 ^M^-1^, respectively. K_SV_ is suggestive of the ligand affinity to DNA and indicates the strength of a given binding interaction. 

Based on the nature of a binding interaction, the fluorescence quenching process is classified into two mechanisms: dynamic (collision) and static (contact). In a dynamic process, fluorophore and quencher are in contact just throughout the excited state (21). In contrast, when the dominant quenching process is static, a complex forms between fluorophore and quencher in the ground state. Unlike the static process, the dynamic mechanism is highly dependent on diffusion. Accordingly, these mechanisms can be differentiated based on their temperature dependence.

With the aim of evaluating the impact of temperature on the quenching constants, the fluorescence experiments were conducted at three different temperatures (298, 303, and 308 K). The Stern-Volmer plots at these temperatures are shown in Figures 2B and 3B. Accordingly, the Stern-Volmer plots of F_0_/F versus concentrations of the quencher were found to be linear which reflects the fact that only a single class of fluorophore, with the identical accessibility to the quenchers, is present in the binding interactions (22). To put it differently, in Amb-ctDNA and Amb-ctDNA-H_1_ interactions only one type of quenching mechanism is presented. When a dynamic quenching mechanism dominates the quenching process, the K_SV_ values increase with increasing temperature. In contrast, when temperature decreases, the value of the static quenching constant decreases, because the stability of the formed complex reduces at high temperatures (23). According to Figures 2B and 3B, it is evident that in both systems, the K_SV _values increase as the temperature is raised, showing that the fluorescence quenching of Amb-ctDNA and Amb-ctDNA-H_1_ systems are controlled by diffusion. 

***Thermodynamic measurements***

The results of fluorescence quenching assays were applied to calculate the values of binding constant (K_b_) using the modified Stern-Volmer equation:

Log [(F_0_-F)/F] = log K_b_ + nlog [Q]                                      (2)

where F_0 _and F are the fluorescence intensities of Amb in the absence and presence of ctDNA (or ctDNA-H_1_), respectively. [Q] is the concentration of quencher (i.e., ctDNA or ctDNA-H_1_) and n is the binding stoichiometry. The calculated values of K_b_ are listed in Table 1. As can be seen, all the binding constants are in the order of 10^8^ indicating a strong interaction between Amb and ctDNA in the absence or presence of the linker histone. 

The values of K_b_ for each system were measured at three different temperatures. Subsequently, the following equations were utilized for expressing the main thermodynamic properties of the binding interactions:

lnK = -∆H^0^ / RT + ∆S^0^ / R                                      (3)

∆G^0^ = ∆H^0^ - T∆S^0^                                      (4)

**Figure 4:**
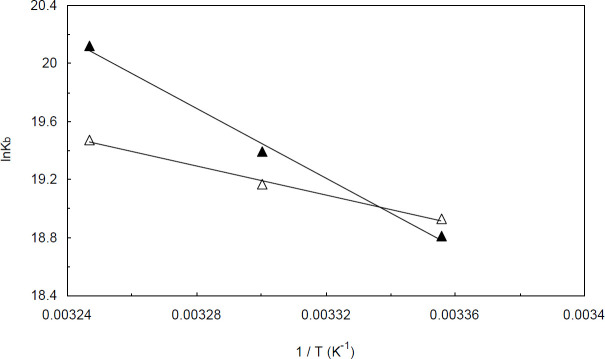
The Van’t-Hoff plots for Amb-ctDNA (closed triangles) and (Amb-ctDNA) linker histone (open triangles)

Where R is the ideal gas constant; T is absolute temperature, and K is the binding constant. ΔH^0^, ΔS^0^, and ΔG^0^ are the changes of binding enthalpy, entropy, and Gibbs free energy, respectively. The slope and intercept of the derived plots from equation 3 were used for approximate determination of the binding enthalpy and entropy changes, respectively (Figure 4). ΔG^0 ^is believed to be the foremost thermodynamic parameter used to describe a binding interaction. The major importance of ΔG^0^ mainly results from the fact that this parameter determines the stability of any given biological complex (24, 25).The numerical value of ΔG^0 ^changes through binding interactions due to the induced conformational changes upon binding. It should be noted that for a given interaction, the values of ΔH^0^ and ΔS^0^ do not change over the short range of temperature, whereas ΔG^0 ^is significantly temperature-dependent and tends to vary with temperature.

Binding constants and thermodynamic parameters for Amb-ctDNA and Amb-(ctDNA-H_1_) interactions are listed in Table 1. The negative values of ΔG^0^ suggest that the binding processes are all exergonic and spontaneous. Furthermore, the results reveal that the binding interactions between Amb and ctDNA/ctDNA-H_1_ are enthalpy-disfavored while the entropy contribution is favored. 

One reliable approach commonly utilized to estimate the acting force in an interaction is to determine the thermodynamic variables of the binding process. It is well-known that four types of forces mainly contribute to non-covalent binding interactions: (1) electrostatic forces, (2) van der Waals forces, (3) hydrogen bonds, and (4) hydrophobic forces. It has been stated that the positive values of ΔH^0^ and ΔS^0^ generally arise from hydrophobic interactions, while negative values of entropy and enthalpy changes are associated with hydrogen bonds and/or Van der Waals forces. Moreover, the negative value of ΔH^0 ^along with the positive value of ΔS^0^ indicates that the major force in the binding process is the electrostatic force (26, 27). Accordingly, the positive values of ΔS^0^ and ΔH^0^ in our experiments allow us to conclude that hydrophobic interactions played the main role in stabilizing both Amb-ctDNA and Amb-(ctDNA-H_1_) complexes. 

***Resonance light scattering (RLS) studies***

**Figure 5:**
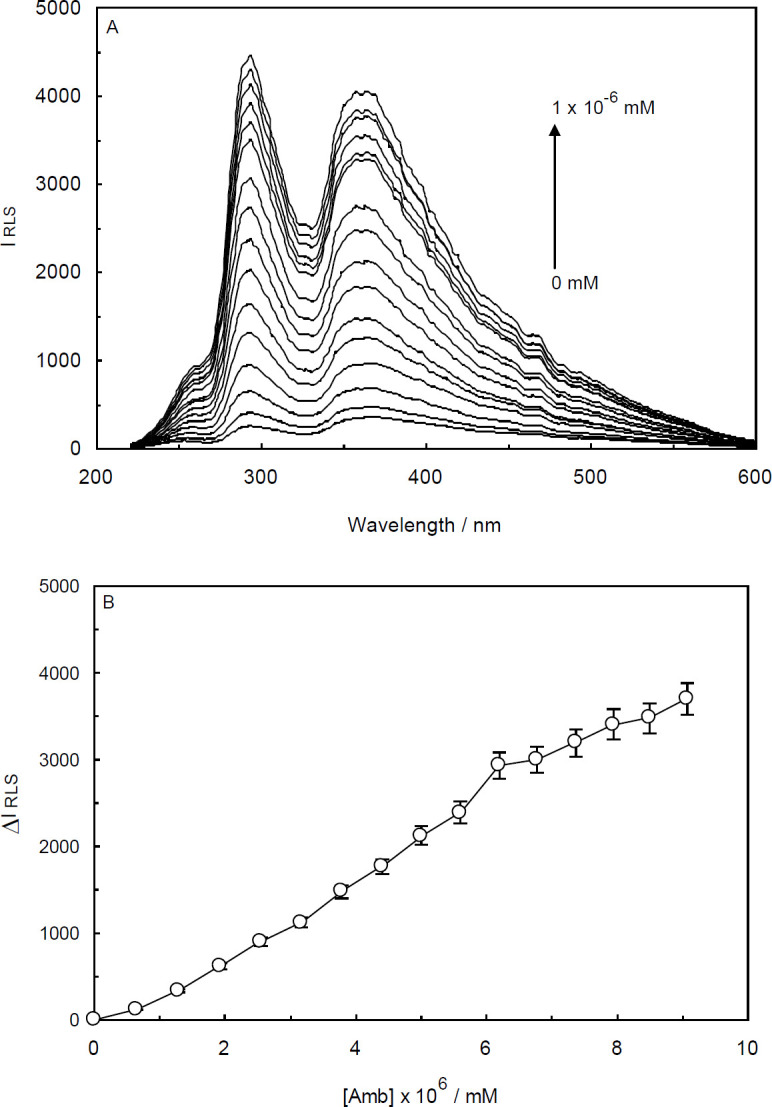
(A) Resonance Light Scattering (RLS) spectra of Ambochlorin (Amb) in the absence and presence of ctDNA. Amb concentration was kept constant at 0.07 mM, while ctDNA concentration was gradually increased from 0 to 1×10-6 mM at T = 298 K and pH 6.8. (B) The curve of ΔIRLS versus the concentration of ctDNA

**Figure 6:**
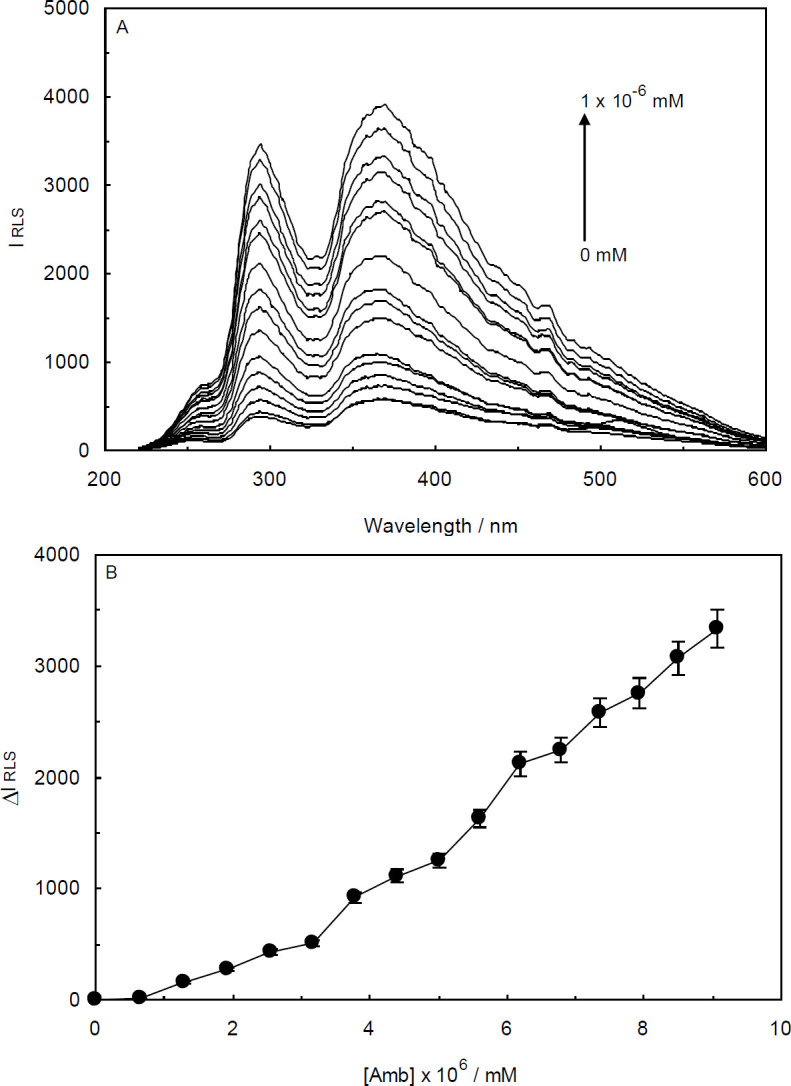
(A) Resonance Light Scattering (RLS) spectra of Ambochlorin (Amb) (0.07 mM) in the absence and presence of various concentrations of ctDNA- linker histone complex. Amb concentration was fixed at 0.07 mM, while the ctDNA-H_1_ concentration was increased from 0 to 1×10-6 mM at T = 298 K and pH 6.8. (B) Curve of ΔIRLS versus the concentration of ctDNA-H_1_

The data collected from the RLS studies are visualized in Figures 5 and 6. As illustrated, Amb presented weak RLS signals with two stronger peaks at about 295 and 375 nm. By adding small aliquots of ctDNA and ctDNA-H_1_ to the cell containing Amb, the scattering spectra displayed progressive and prominent enhancement in the RLS signal intensity which was indicative of the complex formation (28, 29). According to Figures 5A and 6A, the increase in the RLS intensity for Amb-ctDNA interaction are modestly higher than that for the Amb-ctDNA-H_1_ system under the same conditions. In other words, more aggregated complexes were assembled in the absence of the linker histone. 

As can be seen from Figures 5B and 6B, the derived plots from the RLS data were rather linear and the difference resonance light scattering values (which is expressed as ΔI_RLS_ = I_RLS_ − I_0RLs_) were continually and moderately enhanced by increasing the concentrations of ctDNA and ctDNA-H_1_. In the RLS experiments, the concentration of DNA in which the RLS intensity remains steady is considered as DNA saturation concentration. 

***Fluorescence competition studies***

Fluorescence competition studies can provide a revealing insight into the binding mode of ligand–DNA interactions. Although the fluorescence intensity of the probe molecules may be decreased or increased by binding to DNA (30), the fluorescence intensity of DNA is always enhanced by binding to the probe molecules. The current displacement studies were performed using Ethidium bromide (EB) and Acridine orange (AO). EB is a trypanocidal small drug (31) that is known to be a classic intercalator with a high affinity to DNA double-strand molecules. Previous studies showed that EB is able to strongly bind to double-helical RNAs as well (32). EB represents weak fluorescence intensity attributed to its planar aromatic ring. When EB binds to double-strand DNA molecules, the fluorescence intensities of both EB and DNA markedly increase. The presence of a second DNA-binder agent can quench the fluorescence intensity of the EB-DNA complex. AO is a kind of fluorescent cationic dye (33) that has similar fluorescence characteristics as EB. It interacts with DNA intercalately and has poor fluorescence intensity that can be significantly enhanced by binding to DNA.

**Figure 7:**
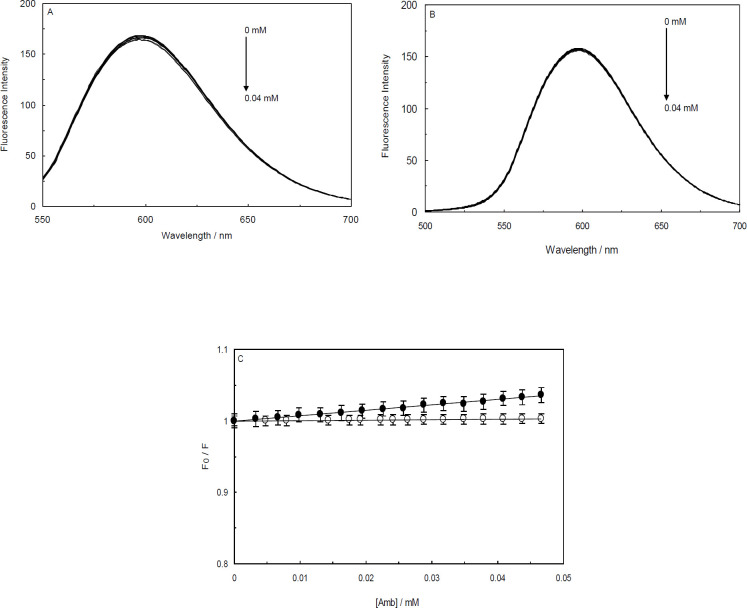
Fluorescence competition studies in the presence of Ethidium Bromide (EB), (A); Fluorescence titration of the EB-ctDNA system by Ambochlorin (Amb), (B); Fluorescence titration of the EB-ctDNA-H_1_ system by Amb, (C); Stern-Volmer plot of relative fluorescence intensity of EB-ctDNA (closed circles) and EB-ctDNA-H_1 _(open circles) in the presence of different concentrations of Amb (0-0.04 mM)

In the current study, Amb was gradually added to a solution containing EB-ctDNA complex and then the data, which were collected by the fluorescence spectrophotometer, were analyzed by plotting the Stern-Volmer relationship (Eq. 1). According to Figure 7A, by increasing the concentration of Amb, a trivial decline in the fluorescence intensities of the EB-ctDNA system was observed. In the case of (ctDNA-H_1_)-EB system, barely any spectral changes were seen by increasing Amb content (Figure 7B). The observations clearly implied that the drug did not displace EB from the complexes. A persuasive argument for this phenomenon is that Amb has different binding sites, and therefore, it does not compete against EB for binding to ctDNA or ctDNA-H_1_. Another possible argument is that Amb might have the same binding sites on ctDNA molecules as EB. However, it can hardly displace EB from the systems, and therefore, Amb cannot bind to ctDNA when the EB molecules are present in the solution. It is evident that Amb has a considerably high affinity to ctDNA (Tables 1A, Table 1B). Thus, we can conclude that Amb interacts with DNA grooves in the absence or presence of the linker histone. As Figure 7C shows, the calculated values of the quenching constants, obtained from Stern-Volmer plots of the Amb-(ctDNA-EB) and Amb-(ctDNA-H_1_-EB) systems, are considerably low (7.55 × 10^2^ and 70 M^-1^, respectively).

**Figure 8:**
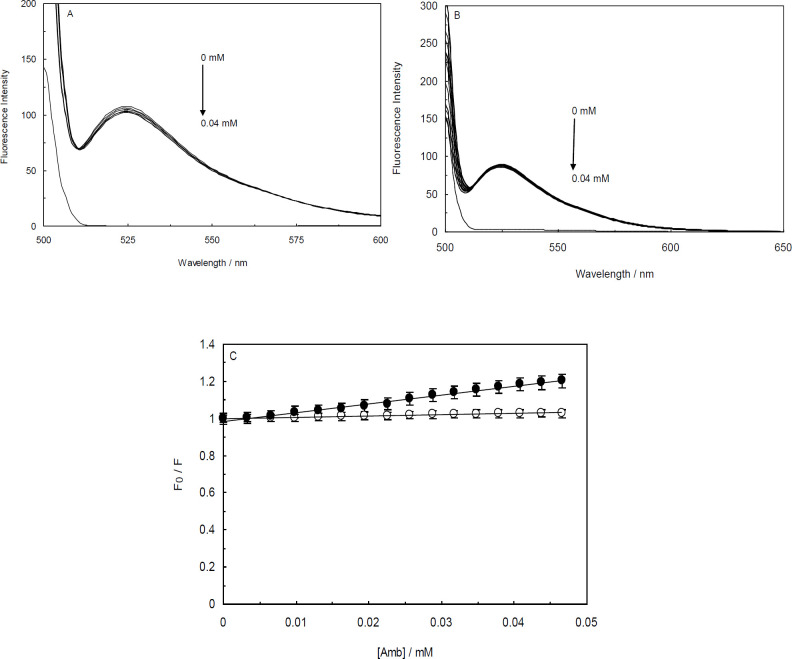
Fluorescence competition studies in the presence of Acridine Orange (AO), (A); Fluorescence titration of AO-ctDNA system by Ambochlorin (Amb), (B); Fluorescence titration of AO-ctDNA-H_1_ system by Amb, (C) Stern-Volmer plot of relative fluorescence intensity of AO-ctDNA (closed circles) and AO-ctDNA-H_1 _(open circles) in the presence of different concentrations of Amb (0-0.04 mM)

Other fluorescence competition experiments were performed to evaluate the validity of the EB displacement studies. This exploration was conducted using the DNA fluorescence probe, AO. According to Figures 8A and 8B, the fluorescence intensities of the AO-ctDNA and AO-(ctDNA-H_1_) systems were quenched marginally in the presence of Amb. The values of K_SV_ for the AO-ctDNA and AO-(ctDNA-H_1_) systems were measured to be 4.75 × 10^3^ and 6.61 × 10^2^ M^-1^, respectively. 

***Ionic strength effect on binding***

Outside or external binding is the electrostatic interaction occurring between the DNA backbone and positively charged groups of ligand molecules. This non-covalent interaction can present as the dominant mode or sub-mode of binding along with other non-covalent modes. Assessing the salt effect on the binding interaction between ligand and DNA is a reliable approach to evaluate the involvement of electrostatic interactions in a DNA-binding process. The analyses are based on the fact that the external bindings are highly sensitive to salt concentrations despite groove binding or intercalation.

**Figure 9:**
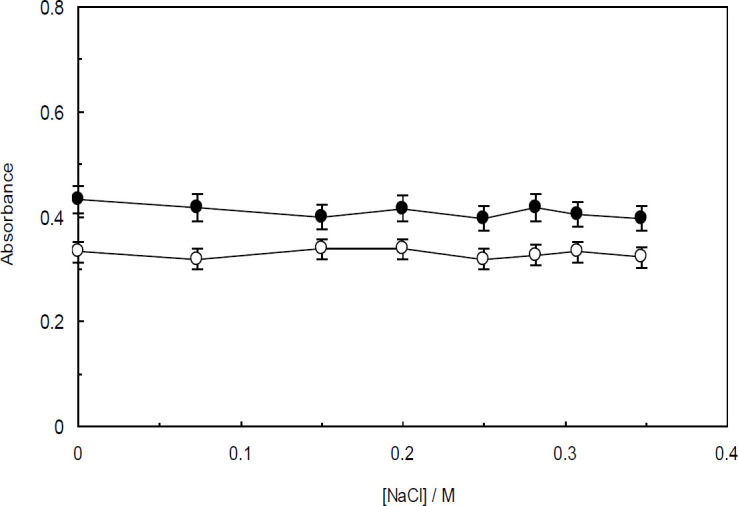
Effect of increasing addition of NaCl (1 M) on the absorbance of Ambochlorin (Amb)-ctDNA (closed circles); and Amb-ctDNA-H_1_ (open circles)

**Figure 10:**
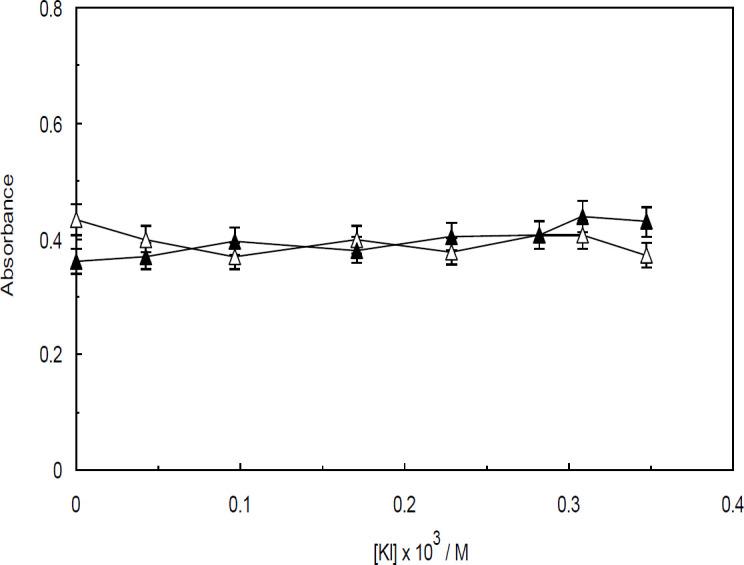
Effect of increasing addition of KI (76.1 mM) on the absorbance of Amb-ctDNA (closed triangles) and Ambochlorin (Amb)-ctDNA-H_1 _(open triangles)

In the experiment designed to investigate the effect of ionic strength on the interaction between Amb and ctDNA in the presence and absence of the linker histone, the UV-visible spectral changes of Amb-ctDNA and Amb-(ctDNA-H_1_) were monitored upon the incremental addition of sodium chloride (NaCl) and potassium iodide (KI). When these strong electrolytes are added to an aqueous solution containing DNA, Na^+^and K^+ ^cations are absorbed by the backbone of DNA. This phenomenon results in a decline in the electrostatic interactions present between the drug and DNA. The effect of increasing concentrations of NaCl and KI on the absorbance spectra of Amb-ctDNA is shown in Figures 9 and 10, respectively. Accordingly, the absorption of Amb-ctDNA is almost unchanged upon increasing addition of NaCl and KI, albeit entirely minor fluctuations can be observed. Similar patterns were observed when KI and NaCl were used to control the ionic strength of the ctDNA-H_1_ solution (Figures 9 and 10). 

***Thermal denaturation studies***

Further investigations on the interactions between Amb and ctDNA were performed using thermal denaturation experiments. DNA thermal denaturation, also known as DNA melting, is a process in which the native DNA molecule (dsDNA) unwinds and dissociates to single strands (ssDNA) upon increasing temperature. The process results in a 30 to 40% rise in the UV absorption of DNA at 260 nm. The temperature at which 50% of the native molecules break and are remodeled into ssDNA is referred to as melting temperature (T_m_). The value of T_m_ can be observed in the vicinity of the transitional point of the DNA melting curve. 

**Figure 11:**
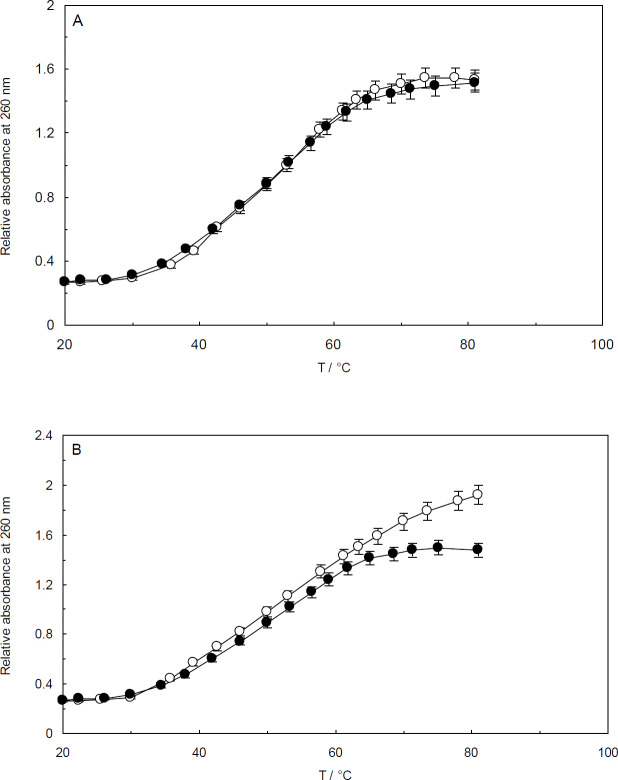
Thermal denaturation assays. The spectra of ctDNA in the absence (closed circles) and presence (open circles) of Ambochlorin (Amb) (A). The spectra of ctDNA linker histone complex in the absence (open circles) and presence of Amb (closed circles) (B). The concentration of Amb was 0.07 mM. CtDNA and ctDNA-H_1_ concentration was 1.3×10^-4^ M

When a ligand binds to DNA, the structure of the DNA double helix can be destabilized or stabilized. Since T_m_ is closely related to DNA stability, the value of T_m _may be affected as a result of binding interactions. In this study, the thermal denaturation process of ctDNA was carried out by monitoring the absorbance of ctDNA and ctDNA-Amb at various temperatures (20–80 °C).To evaluate the role of the linker histone, an investigation was conducted by monitoring the maximum absorbance of the ctDNA-H_1_ complex under the same experimental conditions as for the ctDNA-Amb system. It is evident from Figure 11 that the T_m_ values for ctDNA and ctDNA-H_1_ complex were both around 53^°^C (34, 35). As shown in Figures 11A and 11B, no considerable changes were observed in the T_m _values of ctDNA and ctDNA-H_1_ upon addition of Amb. Therefore, the conformational changes induced by the binding process did not stabilize or destabilize ctDNA and ctDNA-H_1_ complexes. It can be thus concluded that Amb presumably binds to the grooves of ctDNA. This conclusion gives further support to the validity of our other experimental results.

***Viscosity measurement studies***

Viscosity measurement is a hydrodynamic method and one of the most powerful and sensitive techniques for detecting the mode of a binding interaction. When a ligand binds to DNA via electrostatic or groove binding modes of interactions, no or minor changes in the viscosity of DNA may be observed (36). On the contrary, ligand intercalation into DNA base pairs leads to a considerable increase in the viscosity of DNA. The observed rise in the viscosity is due to DNA lengthening induced by intercalators.

**Figure 12:**
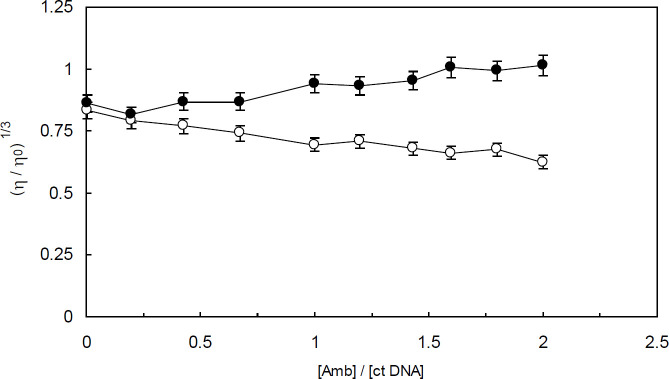
Viscosity measurement studies. The effect of increasing concentration of Amb on the relative viscosity of ctDNA (closed circles); and the effect of increasing concentration of Ambochlorin (Amb) on the relative viscosity of ctDNA-H_1_ complex (open circles). The constant concentration of ctDNA and ctDNA-H1 was 1.3×10^-4^ M

In the current study, the viscosity of ctDNA and ctDNA-H_1_ systems upon adding various concentrations of Amb was visualized by plotting (η / η_0_) ^1/3 ^versus (3) / [ctDNA] or (3) / [ctDNA-H_1_] (Figure 12). As can be seen, the relative viscosity of ctDNA increased slightly upon increasing addition of Amb to the solution. However, the incremental value was not as pronounced as that seen for intercalators, which is suggestive of a groove binding interaction. In the case of ctDNA-H_1_, a minor decline in the relative viscosity was observed. The observed behavior pattern can be correlated to the groove binding mode of interaction.

***Circular dichroism spectroscopy***

**Figure 13:**
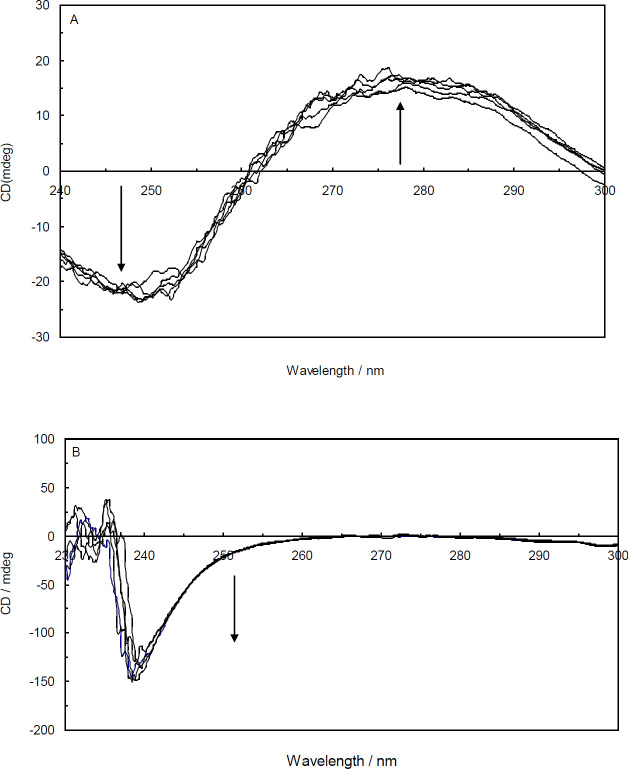
UV-CD spectra of ctDNA in the absence and presence of Amb (A); UV-CD spectra of ctDNA-H1 in the absence and presence of Ambochlorin (Amb) (B). (arrow shows the circular dichroism (CD) changes upon increasing concentration of Amb)

Binding interaction between a ligand and DNA can induce changes in the structure of DNA double helix. Circular dichroism (CD*) *spectroscopy is an accurate and sensitive method extensively used for monitoring the conformational changes of DNA and protein molecules during binding interactions. Generally, the intercalating agents unwind and lengthen DNA to adjust the sugar-phosphate backbone for setting their aromatic ring (37). As a consequence, intercalating agents cause a noticeable alteration in the DNA conformation. On the other hand, external and groove binding agents induce no or minor changes in the DNA conformation (38). According to Figure 13A, the CD spectrum of ctDNA in the absence of Amb showed typical characteristics of the B-form of DNA. The positive and negative peaks around 276 nm and 248 nm are attributed to the base stacking and DNA helicity, respectively. Any changes in the position or intensity of the CD spectral bands during a binding interaction are connected with the DNA conformational changes. Upon incremental addition of Amb to ctDNA, the intensities of both negative and positive bands were enhanced on a small scale and no apparent shifts were observed. The minor increment in the negative ctDNA bands indicates an inconsequential rise in ctDNA helicity. The induced slight helicity increase made ctDNA trivially tighter as a result of local perturbations (39).

Similar experiments were conducted when the ctDNA-H_1_ was titrated by various concentrations of Amb. It can be seen from Figure 13B that the ctDNA-H_1_ in the absence of the drug provided a negative peak at about 240 nm which is related to the protein part of the complex. The CD spectrum of ctDNA is different from that of the ctDNA-H_1_ because ctDNA conformation is significantly affected by the linker histone. In the presence of various concentrations of Amb, the ellipticity of ctDNA-H_1_ at 240 nm becomes more negative with marginal changes in the position of peaks. The results suggest that when Amb is bound to the ctDNA-H_1_ modest conformational changes take place in the ctDNA. 

***Stopped-flow circular dichroism***

Evaluating the kinetics of binding interaction is a highly beneficial approach used to obtain practical details for characterizing a binding process. The stopped-flow analysis is proven to be a highly sensitive and accurate method for monitoring the binding process and determining the binding kinetic constants. The sensitivity of stopped-flow methods is widely dependent on the detector. A stopped-flow accessory can be combined with optical techniques, such as absorbance, fluorescence, and circular dichroism spectroscopy to collect information regarding the kinetics of biological systems. The stopped-flow CD is a widely used technique to investigate the DNA-ligand interactions. It is also frequently utilized to study the conformational changes of proteins (40). In the current study, the stopped-flow CD technique was applied to clarify the details on the binding interaction between ctDNA and Amb in the absence and presence of the linker histone. The time-dependent changes of ctDNA and ctDNA-H1 conformations upon binding to Amb were evaluated by monitoring the changes of CD signals against time. In order to achieve the best desired results, the experiments were performed at the wavelength in which ctDNA and ctDNA-H1 had the highest CD signals.

The obtained raw data from the experiments were fitted and normalized by using the following ﬁrst-order exponential equation:

$y\left(t\right)=y_{0}+{\mathrm{Ae}}^{-\frac{t}{t_{1}}}$                                      (5)

where y(t) is the observed CD signal at time t, y_0_ and A are fitting parameters (41) and t_1 _is the characteristic binding time. The value of the binding rate can be obtained by calculating 1/t_1_. 

**Figure 14:**
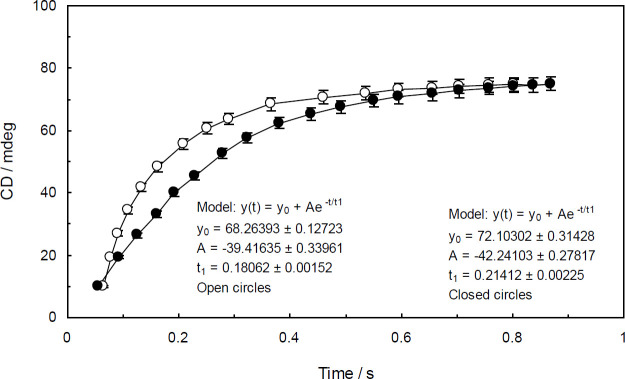
Stopped-flow CD kinetic traces for Amb-ctDNA (closed circles) and Ambochlorin (Amb)-ctDNA-H1 (open circles) binding interactions. The solutions were mixed in a 1:1 volume ratio. The circular dichroism (CD) signals of ctDNA and ctDNA-H1 (1.3×10-4 mM) were enhanced upon binding to Amb (0.07 mM). The ﬁrst-order exponential decay model was applied to fit the raw data

In the present study, the values of t1 for the binding interaction between Amb and ctDNA and ctDNA-H1 were found to be more than 150 milliseconds. The characteristic binding time of Amb-ctDNA was shorter than that of Amb-ctDNA-H1 by an approximate difference of 33.5 milliseconds. The derived plots from Eq.5 are visualized in Figure 14. In the present stopped-flow experiment, by applying a CD detector the conformations of ctDNA and ctDNA-H1 were monitored for the duration of the binding processes. When the flows were stopped, ctDNA and ctDNA-H1 conformations began to change after a short delay. The binding stopped-flow kinetic plots for Amb-ctDNA and Amb-ctDNA-H1 were rather similar in shape. As can be seen, the CD signals of both ctDNA and ctDNA-H1 intensified upon binding to Amb. However, the kinetic proﬁle of Amb-ctDNA-H1 presented a shallower rising compared with that of Amb-ctDNA, which is indicative of the fact that the kinetics of the binding interaction between Amb and ctDNA was influenced by the linker histone. 

***Comparison of the interaction of Amb with ss ctDNA and ds ctDNA***

Comparing the fluorescence quenching effect of denatured and native DNA molecules on the fluorescence intensity of a ligand gives us beneficial insights into the binding mechanism. It is commonly investigated that when a ligand intercalates into DNA, the fluorescence quenching effect of native ds ctDNA on the ligand intensity should be larger than that of ss ctDNA. In contrast, the ligands that bind to the grooves of DNA undergo a stronger quenching effect by ss ctDNA titration than by native DNA. In the outside binding mode, the quenching intensity by the ss ctDNA and native ds ctDNA are equal (42).

**Figure 15:**
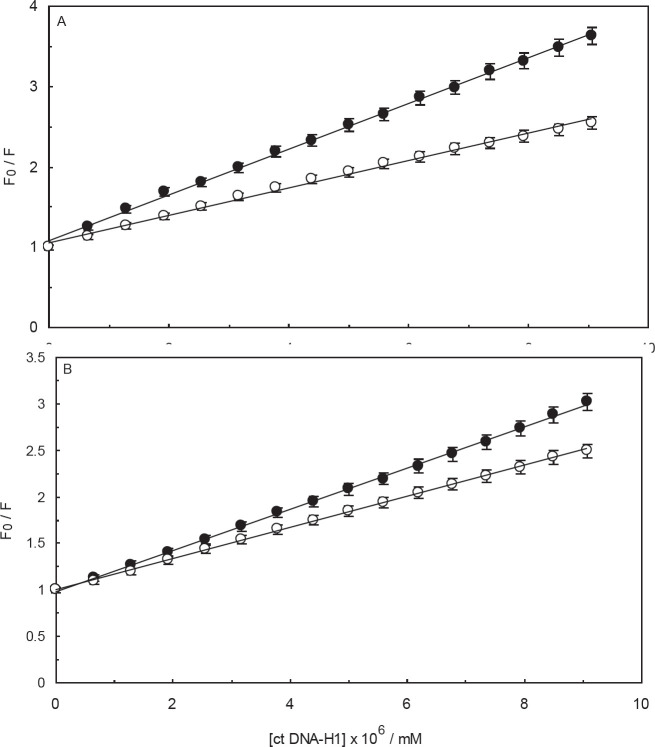
Comparison of quenching effect of ss ctDNA(closed circles) and ds ctDNA (open circles) on Ambochlorin (Amb) (A); The comparison of quenching effect of ss ctDNA-H1 (closed circles) and ds ctDNA-H1 (open circles) on Amb (B). The concentration of ctDNA and ctDNA-H_1_ systems was 1.3×10^-4^ mM

As Figure 15A shows, the fluorescence intensity of Amb was considerably quenched by adding both ss ctDNA and native ds ctDNA. The K_SV _values for Amb-ss ctDNA and Amb-ds ctDNA were found to be 2.84×10^8^ and 1.46×10^8^ M^-1^, respectively indicating that Amb had a higher affinity to ss ctDNA. Therefore, we can conclude that Amb interacts with the native ctDNA in grooves.

Similar fluorescence tests were performed in the presence of the linker histone. The results showed that (ss ctDNA)-linker histone had a stronger quenching effect on Amb than on the ds ctDNA-H_1_ (Figure 15 B). The values of K_SV _for the (ss ctDNA)-linker histone-Amb and (ds ctDNA-H_1_)-Amb were found to be 2.21×10^8 ^and 1.67×10^8 ^M^-1^, respectively suggesting that Amb preferred ss ctDNA-H_1_ rather than ds ctDNA-H_1_. Thus, the nature of the Amb-(ctDNA-H_1_) interaction is groove binding. In addition, the obtained values of K_SV _confirm that Amb has a higher affinity to ctDNA in the presence of the linker histone.

***Molecular modeling studies***

**Figure 16:**
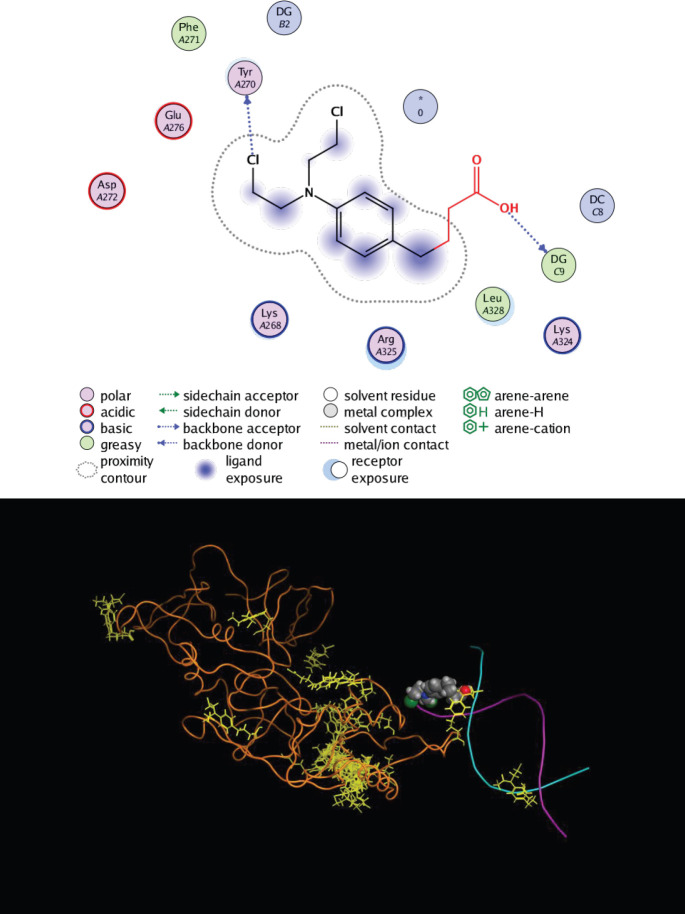
Docking simulation of the binding interaction between Ambochlorin (Amb) and ctDNA (A). As shown, Amb was inserted into the minor grooves of ctDNA. The complex was stabilized by hydrophobic forces and hydrogen bonds. Docking result of Amb-ctDNA-H_1_ binding interaction (B). Amb molecules have been shown as yellow in docking. Amb was located in the major groove of DNA while interacting with the linker histone as well. The dominant forces in the binding interaction were hydrophobic

Molecular modeling investigations play an important role in predicting and evaluating the mechanism and characteristics of the binding interactions between DNA and ligand molecules (43, 44). With the aim of providing a more detailed understanding of the interactions between Amb and ctDNA in the absence and presence of the linker histone, the binding interactions were simulated by the MOE software. Amb molecules were separately docked by ctDNA and ctDNA-H_1_. The lowest energy-ranked conformers of Amb-ctDNA and Amb-ctDNA-H_1_ were selected and then employed for further analysis (Figures 16A and B). The docking result of Amb-ctDNA binding interaction clearly indicated that Amb was inserted into the minor groove of ctDNA with the lowest binding free energy of -10.34 kcal.mol^-1^. Further analysis revealed that Amb interacted with the guanine bases of double helix strands in the minor grooves (45). It was also found that the hydrophobic forces and hydrogen bonds are the dominant forces that stabilized Amb-ctDNA complex. Another docking run was performed to investigate the interactions between Amb and cDNA in the presence of the linker histone. The docking result of the Amb-(ctDNA-H_1_) interaction revealed that Amb was located in the major groove of DNA when interacting with the linker histone. More analysis revealed that the main force that stabilized the formed complex was hydrophobic interaction. The binding energy for this interaction was found to be -10.72 kcal.mol^-1 ^which is slightly lower than Amb-ctDNA interaction.

## Discussion

In the last decades, investigations on the DNA-drugs interactions have attracted increasing attention. In the present study, the binding interaction between Amb and ctDNA in the presence and absence of H_1_ as binary and ternary systems has been investigated.

Fluorescence spectroscopy is a powerful applicable method widely employed to analyze the interactions between DNA and other DNA-binding molecules, such as drugs, proteins, and ion molecules (46). In the current study, the high values of K_SV_ for these interactions suggested a high binding affinity between Amb and ctDNA in the absence or presence of the linker histone. However, the greater K_SV_ value for the Amb-ctDNA-H_1_ system implied that Amb had a higher affinity to ctDNA-H_1_ compared with the naked ctDNA. Based on the above statements, we infer that the fluorescence quenching of Amb-ctDNA and Amb-ctDNA-H_1_ systems are diffusion-dependent and dominated by a dynamic process.

The formation of a complex between a ligand and a receptor generally requires both bond breakage and formation. The difference between the energy released upon bond-breaking and used upon bond-forming determines whether the binding process is endothermic or exothermic. The change of energy in a binding process is reflected in terms of binding enthalpy changes (47, 48). According to thermodynamic measurements, the values of binding enthalpy for Amb-ctDNA and Amb-ctDNA-H_1_ interactions are positive indicating that both binding processes are endothermic.

Determination of the type of forces involved in a binding interaction is an indirect approach to predict the binding mode of a DNA-ligand interaction. In the intercalation mode, van der Waals forces generally stabilize the complex, while in the minor groove binding the complex is mostly stabilized by the hydrophobic interactions. Therefore, we can infer that Amb probably interacts with ctDNA and ctDNA-H_1_ complex through minor grooves.

Since the RLS spectroscopy is very sensitive to molecular aggregations, it can be effectively utilized to study the binding interaction between DNA and its ligands (49). In the RLS phenomenon, the extent to which a particle may absorb and then scatter light is based on its size and shape. Accordingly, the observed results of RLS studies suggest that under our experimental conditions the saturation concentration of ctDNA and ctDNA-H_1_ with Amb (0.07 mM) were higher than the maximum applied concentration.

The competitive interaction technique is an applicable method in which DNA fluorescence probes are employed in competitive displacement experiments, and sensitive fluorophore molecules bind to DNA usually with high affinity (50). The obtained results of competition studies represented using AO were completely in line with those using EB. In summary, the fluorescence competitive studies evidently showed that Amb interacts with the grooves of ctDNA in the absence or presence of the linker histone.

On the basis of the provided data from the ionic strength effect on binding, we conclude that no considerable electrostatic forces contribute to the complex formation between Amb and ctDNA/ctDNA-H_1_.

It has been recognized that intercalation of small molecules to DNA is likely to increase T_m_ by about 5–8 °C (51). The considerable rise in the T_m_ value is due to the fact that the stability of DNA molecules generally increases when a ligand binds to it intercalately. On the contrary, non-intercalation modes of binding (i.e., groove and outside binding) lead to no or trivial changes in the T_m_ values. Since the T_m _value of ctDNA almost remained unchanged in the presence of the linker histone_,_ it is reasonable to deduce that the presence of the linker histone has no perceptible impact on ctDNA stability.

It should be noted that different behavior patterns of ctDNA viscosity changes observed in the absence and presence of the linker histone, i.e., minor positive versus minor negative changes, are mainly due to ctDNA conformational changes induced by the linker histone.

Generally, the data collected by the CD tests showed that Amb did not induce remarkable conformational changes in ctDNA which is a characteristic of the groove binding agents (52). Regarding the fact that the spectral changes were not as pronounced as expected for the intercalation mode, we can thus conclude that Amb is probably bound to the ctDNA-H_1_ complex through interacting with ctDNA grooves.

Based on the data gathered by the stopped-flow CD experiments, we can infer that the rate of the complex formation between Amb and ctDNA was marginally slowed down in the presence of the linker histone (53). Moreover, the results indicate that the linker histone affects the kinetics of the binding interaction between Amb and ctDNA. In general, the results obtained by the experimental analysis were entirely verified and complemented by the molecular modeling studies. Based on the results obtained by docking studies, it can be concluded that the linker histone changed the binding sites of Amb from ctDNA minor grooves to the major grooves.

**Table 1:** The thermodynamic parameters and binding constants for Amb-ctDNA (A) and Amb-ctDNA-linker histone (B) at three different temperatures

T	K_b_ / M^-1^	ΔG^0^ / kJ. mol^-1^	ΔH^0^ / kJ. mol^-1^	ΔS^0^ / J. mol^-1^K^-1^
				
298	(1.46 ± 0.03) × 10^8^	-46.58		
				
303	(1.97 ± 0.04) × 10^8^	-48.11	99.89	491.37
				
308	(5.48 ± 0.03) × 10^8^	-51.53		
T	K_b_ / M^-1^	ΔG^0^ / kJ. mol^-1^	ΔH^0^ / kJ. mol^-1^	ΔS^0^ / J. mol^-1^K^-1^
				
298	(1.67 ± 0.02) × 10^8^	-46.91		
				
303	(2.13 ± 0.03) × 10^8^	-48.13	41.18	295.47
				
308	(2.85 ± 0.03) × 10^8^	-49.85		

## Conclusion

The current study was conducted to have an insight into the binding interaction between Amb and ctDNA. We further investigated whether the presence of the linker histone may affect the interaction between Amb and ctDNA. To obtain a detailed understanding of the nature of interaction, different spectroscopic and molecular modeling techniques were employed. In addition, the binding kinetics of the interactions was investigated using the stopped-flow CD technique. The spectroscopic experiments revealed that Amb can bind to both naked ctDNA and ctDNA-H_1_ complex with different affinities. The binding constants were found to be of the order of 10^8^ M^-1^ which is suggestive of the considerably high affinity of Amb to ctDNA and ctDNA-H_1_ complex. However, Amb showed a higher affinity to ctDNA in the presence of the linker histone_. _The results indicated that the quenching mechanism in both interactions was governed by a dynamic process. The thermodynamic parameters for the binding interactions showed that both binding processes were spontaneous and endothermic. The formed complexes between Amb and ctDNA/ctDNA-H_1_ were mainly stabilized by the hydrophobic interactions, and no electrostatic forces contributed to the complex formation. The results of fluorescence competition, thermal denaturation, ligand tendency toward native or denatured ctDNA, and CD spectroscopy studies were entirely consistent with each other indicating that Amb interacted with ctDNA through grooves in the absence or presence of the linker histone. The viscosity experiments also provided further support for this finding. Furthermore, it was found that the binding interactions did not induce remarkable changes in ctDNA and ctDNA-H_1_ conformations. The obtained data by the stopped-flow CD experiments showed that the linker histone affects the kinetics of the binding interaction between Amb and ctDNA. The rate of the complex formation between Amb and ctDNA was slightly slowed down in the presence of the linker histone. It was also shown that Amb can interact with the linker histone. The results of the experimental analysis were entirely verified by our molecular modeling studies.

 The study revealed that Amb interacted strongly with ctDNA in the absence or presence of the linker histone. However, the binding strength between ctDNA and Amb enhanced in the presence of the linker histone. On the basis of our experimental results, we conclude that some binding properties of the binding interaction between Amb and ctDNA slightly changed in the presence of the linker histone. It was also suggested by docking analysis that the presence of the linker histone may change the binding site of Amb on ctDNA from minor to major grooves.

## Authors’ Contributions

AAK and JC designed the research study. AAK, PM, MD, MM, and MP performed the research and collected the data. ME, MAL, and JC analyzed the data. AAK and MRS wrote the initial draft of the manuscript. MRS and JC revised the manuscript. All authors discussed the results and contributed to the final manuscript.

## Authors’ Contributions

AAK and JCH designed the research study. AAK PM, MD, MM, and MP performed the research and collected the data. ME, MAL, and JCH analyzed the data. AAK, and MRS wrote the initial draft of the manuscript. MRS and JCH revised the manuscript. All authors discussed the results and contributed to the final manuscript.

## Ethical Statement

There are none to be disclosed.

## Funding

The financial support of the Research Council of the Mashhad Branch, Islamic Azad University and Mashhad University of Medical Sciences are appreciated and acknowledged.

## Conflicts of Interest

The authors declare no conflicts of interest regarding this article.
